# Antibodies Against Lysophosphatidic Acid Protect Against Blast-Induced Ocular Injuries

**DOI:** 10.3389/fneur.2020.611816

**Published:** 2020-12-15

**Authors:** Peethambaran Arun, Franco Rossetti, James C. DeMar, Ying Wang, Andrew B. Batuure, Donna M. Wilder, Irene D. Gist, Andrew J. Morris, Roger A. Sabbadini, Joseph B. Long

**Affiliations:** ^1^Blast-Induced Neurotrauma Branch, Walter Reed Army Institute of Research, Silver Spring, MD, United States; ^2^Division of Cardiovascular Medicine, Lexington VA Medical Center, College of Medicine, University of Kentucky, Lexington, KY, United States; ^3^Department of Biology, San Diego State University, San Diego, CA, United States

**Keywords:** blast exposure, eye injury, ocular dysfunction, lysophosphatidic acid, anti-LPA antibody, rat, retina

## Abstract

Exposure to blast overpressure waves is implicated as the major cause of ocular injuries and resultant visual dysfunction in veterans involved in recent combat operations. No effective therapeutic strategies have been developed so far for blast-induced ocular dysfunction. Lysophosphatidic acid (LPA) is a bioactive phospholipid generated by activated platelets, astrocytes, choroidal plexus cells, and microglia and is reported to play major roles in stimulating inflammatory processes. The levels of LPA in the cerebrospinal fluid have been reported to increase acutely in patients with traumatic brain injury (TBI) as well as in a controlled cortical impact (CCI) TBI model in mice. In the present study, we have evaluated the efficacy of a single intravenous administration of a monoclonal LPA antibody (25 mg/kg) given at 1 h post-blast for protection against injuries to the retina and associated ocular dysfunctions. Our results show that a single 19 psi blast exposure significantly increased the levels of several species of LPA in blood plasma at 1 and 4 h post-blast. The anti-LPA antibody treatment significantly decreased glial cell activation and preserved neuronal cell morphology in the retina on day 8 after blast exposure. Optokinetic measurements indicated that anti-LPA antibody treatment significantly improved visual acuity in both eyes on days 2 and 6 post-blast exposure. Anti-LPA antibody treatment significantly increased rod photoreceptor and bipolar neuronal cell signaling in both eyes on day 7 post-blast exposure. These results suggest that blast exposure triggers release of LPAs, which play a major role promoting blast-induced ocular injuries, and that a single early administration of anti-LPA antibodies provides significant protection.

## Introduction

Exposure to blast overpressure waves is implicated as the major cause of ocular injuries and resultant visual dysfunction in veterans involved in recent combat operations (1). Although personnel are issued protective goggles, eye injuries nevertheless occur due to non-compliance, blast wave penetration through goggles, or goggles being dislodged (2). Between 2006 and 2009, 20 (43%) of 46 combat-veteran in-patients with blast-induced traumatic brain injury (TBI) had significant closed-eye injuries (3). Brain visual signal processing centers (e.g. optic tracts) are also possibly directly perturbed by the blast waves (4). Both clinical and preclinical studies evaluating effects of low-level single and repeated blast exposures have shown significant pathological changes in the ocular system (5, 6). Despite the difficult lifelong disability that loss of vision represents, there have been only a few animal studies assessing blast injuries to the eyes and visual system (7–12) and very few have evaluated drug therapies (13–17). No effective countermeasure has been developed to date.

Using a compressed air-driven shock tube, it has been shown that a single blast exposure in rats can induce cellular apoptosis in the optic nerve, the ganglion layer, and inner nuclear layers of the retina (18–20). A follow-up study demonstrated that single as well as repeated low-level blast exposures trigger increases in the cellular apoptosis marker, caspase-3, and the pro-inflammatory marker, cluster of differentiation 68 (CD68), in the optic nerve (6). Single and repeated low-level blast exposures also greatly increased glial fibrillary acidic protein (GFAP) in the retina (6). Using a blast simulation in which an air-jet is generated by a modified paintball gun that is pointed directly at the eyes of mice, it was found that the exposure caused retinal detachments, large retinal pigment epithelium vacuoles, regional rod photoreceptor cell death, and glial cell reactivity (21). In that study, immune infiltrate was detected throughout the eyes after the insult. These results suggest that blast exposure caused cellular inflammation in the peripheral and central ocular system and therapies which can counter these pro-inflammatory responses might be effective in protecting the ocular system. Glial cell activation and hyperphosphorylation of *Tau* protein, which are known to induce neuronal degeneration, were also observed in the retina up to several days after blast exposure (11). Thus, therapies which can inhibit the hyperphosphorylation of *Tau* protein might also be protective against retinal neurodegeneration. Our laboratory has also shown similar acute and sub-acute changes in ocular functions and retina and brain visual signal processing center (i.e., optic tract) pathologies in rats after single blast exposure using a compressed air-driven shock tube (12).

Lysophosphatidic acid (LPA; 1-acyl-*sn*-glycerol 3-phosphate), which is reported to play major roles in stimulating inflammatory processes (22, 23), is a bioactive metabolite of phospholipids (i.e., phosphatidyl choline) generated by the sequential actions of phospholipase A2 and then lysophospholipase D (autotaxin) within activated platelets, astrocytes, choroidal plexus cells and microglia in response to injuries. LPA species with both saturated fatty acids and polyunsaturated fatty acids have been identified in various biological fluids including plasma, serum and cerebrospinal fluid (CSF). LPA can mediate a series of signaling events (e.g. adenylate cyclase inhibition and endoplasmic reticulum Ca^2+^ mobilization) through specific G-protein coupled LPA receptors. Following central nervous system (CNS) injury, ischemia, or related events that damage the blood-brain barrier, LPA-like activity is increased within the CSF and LPA receptor modulation occurs in different regions of the brain (24, 25). In addition to inducing neuropathic pain and demyelination, LPA can stimulate astrocytic proliferation, inflammation and, depending upon its concentration, can promote death of neurons by apoptosis or by necrosis at high levels (26–32). LPA is reported to be involved in stimulating *Tau* protein phosphorylation through activation of glycogen synthase kinase-3β and protein kinase A leading to neurite retraction in multiple neuronal cell lines (33, 34).

No studies have been carried out to determine the involvement of LPA in blast wave induced eye injuries; however, evidence for a negative impact of LPA on retinal health is strong. Intravitreal injection of LPA leads to a decreased microvasculature in the retinas of rat pups (35). Additionally, using retinal ganglion cells in culture, it has been shown that LPA receptor1 is likely involved in retinal cell degeneration in hyper-oxygenation induced retinopathy of prematurity (36). In an embryonic chick visual system model, introduction of LPA causes a dose-dependent growth cone collapse of cultured retinal neurons (37). Significantly elevated levels of LPAs were detected in vitreous humor samples from the eyes of patients with retinal vein occlusion and were positively correlated with those of pro-inflammatory cytokines, suggesting a role of LPAs in exacerbating secondary pathologies associated with the condition, e.g. macular edema (38).

Increased LPA levels and differential expression of LPA receptors have been implicated in neurotrauma and neuropathic pain (24, 39, 40). Different LPA species were found to be elevated in the CSF of patients with TBI unrelated to blast, due to severe closed-head concussions (e.g. motor vehicle accidents and falls) (24). The levels were significantly higher 24 h after injury and returned to normal levels by day 5, the only two time points evaluated (24). Multiple LPA species were elevated at 3 h and returned to normal levels by 14 h in the CSF of mice subjected to controlled cortical impact (CCI) induced TBI, revealing that extracellular LPAs rapidly increase after brain injury (24). Intravenous administration of the antibodies raised against LPAs significantly improved neuropathology and function after TBI and spinal cord injury in experimental animals, demonstrating that the acutely increased extracellular LPAs are deleterious to the central nervous system and that therapeutic strategies to scavenge these LPAs can be highly beneficial (24, 40). Likewise, intranasal administration of LPA antibodies was found to be advantageous in an animal model of CCI-induced TBI and reduced the symptom of mechanically induced neuropathic pain (allodynia) (39). In the present study, using an Advanced Blast Simulator (ABS), which generates high fidelity blast waves closely resembling those encountered with free field explosions, we have evaluated the efficacy of a single intravenous administration of LPA antibodies for protection against blast-induced ocular injuries.

## Materials and Methods

### Animals

Research was conducted under an animal use protocol approved by the Institutional Animal Care and Use Committee at Walter Reed Army Institute of Research in a facility accredited by the Association for the Assessment and Accreditation of Laboratory Animal Care-International in compliance with the Animal Welfare Act and other federal statutes and regulations relating to animals and experiments involving animals and adheres to principles stated in the Guide for the Care and Use of Laboratory Animals, NRC Publication, 2011 edition. Male Sprague Dawley rats, 9-10 weeks old that weighed 300–350 g (Charles River Laboratories, Wilmington, MA) were housed at 20–22°C (12 h light/dark cycle). Rats were given free access to nutritious rat chow (Prolab IsoPro RMH 3000 from LabDiet, St. Louis, MO) and water *ad libitum*.

### Primary Blast Exposure

The ABS described previously was used for the study (41, 42). For blast exposure, the rats were anesthetized with 4% isoflurane for 8 min and secured in a longitudinal (i.e., rat facing the oncoming shockwave) prone orientation in the test section of the ABS. To produce moderate TBI in rats in these experiments, we used Valmax membranes yielding peak positive static pressures of ~19 psi with a positive phase duration of 4–5 ms. Group sizes for all subsequent outcome measures were six animals each (*n* = 6).

### LPA Measurements

For acute LPA measurements in the plasma, the rats were euthanized at 1 and 4 h post-blast by drawing blood through cardiac puncture under isoflurane anesthesia. Blood was collected in vacutainer tubes (Becton, Dickinson and Company, Franklin Lakes, NJ) containing ethylenediaminetetraacetic acid as the anticoagulant and the plasma separated by centrifugation was used for LPA measurements. The plasma samples were stored at −80°C until analysis. LPA measurements were carried out as described earlier (43). Briefly, lipids were extracted from plasma using acidified organic solvents. Different LPA species were measured by Ultra High Performance Liquid Chromatography (UHPLC) coupled electrospray ionization tandem mass spectrometry using an AB Sciex 6500 Q-Trap mass spectrometer operated in multiple reaction monitoring mode to identify the molecules as based on their specific precursor and product ion pairs. 17:0 LPA was used as an internal standard. Thus, plasma LPA values were reported as μmoles per liter.

### Intravenous Administration of LPA Antibody

One hour after blast exposure, the rats were treated intravenously with either murine anti-LPA monoclonal antibody (504B3, 25 mg/kg) or isotype-matched control antibody (IgG2b, 25 mg/kg) under isoflurane anesthesia in sterile conditions. Sham control rats received the same volume of saline. The antibodies were provided by Lpath Inc. (San Diego, CA).

#### Visual Acuity Testing (Optokinetics)

At 2 days before (baseline) and days 2 and 6 after blast exposure, visual acuity (VA) was tested using an optokinetics device (OptoMotry unit; Cerebral Mechanics Inc., Alberta, Canada). The conscious rat was placed on a pedestal inside a chamber, where it was surrounded on all four sides by LCD monitors that project a virtual rotating black and white bar pattern having a starting spatial frequency at which rats should easily see, e.g. 0.04 line-cycles/degree at 100% contrast. The bar's rotation speed was then increased stepwise to gradually narrow the appearance of their width. Contribution of each eye was resolved by driving the pattern's rotation in opposite directions, e.g. clockwise for the left side (44). Eye pursuits were judged by reflexive movements (side flicks) of the head, which were aligned with the direction of the stimulus rotation. Visual acuity thresholds, as reported in line-cycles/degree, were found by iterations of the spatial frequency of the bar pattern, until head tracking movements were no longer exhibited. The acuities can be measured with a 97% accuracy of 0.01. Normal young Sprague Dawley rats were reported to have visual acuities of 0.5 (45); however, we found as they age, rats fall closer to 0.30.

#### Electroretinography

At 1 day before (baseline) and days 3 and 7 after blast exposure, retinal responses were assessed by full field flash electroretinography (ERG) utilizing an ERG instrument (Color Dome Full Field Ganzfeld unit; Diagnosis LLC, Lowell, MA). Rats were dark adapted overnight (≥16 hours). While working under photography grade red-safelights, animals were placed under continuous 2–3% isoflurane anesthesia via a nose cone device. Pupils were dilated with 0.5% (w/v) tropicamide and 2.5% (w/v) phenylephrine drops in buffered saline. As an anesthetic, each eye received proparacaine drops. Ground electrodes were fixed to the tail, reference inside the mouth, and recording gold-loops (contact lens style) against each eye's cornea with a conducting cushion of methyl cellulose solution. While under darkness, the rat's eyes were presented with an eight step series of white light flashes of increasing illumination, i.e., 0.1–10 cd.s/m^2^ with a 5 ms duration and 30–60 s interval (12). This data yielded scotopic ERG response curves. Peak amplitudes of signaling response wave forms, as recorded in μ-volts, were used to judge the status of retinal rod photoreceptor (A-wave) and bipolar (B-wave) neurons. External influences that can alter the ERGs were minimized, such as environmental electrical noises, dark-room light leaks, and body temperature fluctuations (46). Rats were allowed to recover for at least 3 h under dim room light conditions, to help prevent retina photo-damage from excessive light stimulation until the pupillary reflex returns. Since the ERG signals can vary among rats, the percentage change from each experimental subject's own baseline values were used for measurements after blast exposure.

#### Histopathology and Immunohistochemistry

Paraformaldehyde (PFA) fixed eyes collected on day 8 after blast exposures were processed by FD Neurotechnologies, Inc. (Ellicott City, MD) for histopathological (H&E staining) and immunohistochemical staining. Eyes were cut as a single horizontal section through the central pupil to optic nerve axis as described by our laboratory (12). Sections were stained with H&E to determine gross morphological changes in the retina, including neuronal cell loss and detachments. Distinct neuronal layers of the entire retina (from optic nerve head to periphery) were examined, and included ganglion, bipolar, and rod photoreceptor cells (12). Injury scores for the retina were ranked on an ordinal scale by highly trained reviewers blinded to treatments using values of 1–6, representing no, slight, mild, moderate, severe, and catastrophic levels of injury, respectively. After H&E staining, the remaining paraffin sections were used for immunohistochemistry to detect activated Müller giant glial cells and their extensions that highly express glial fibrillary acidic protein (GFAP) in the retina using rabbit polyclonal antibodies against GFAP (Abcam, Cambridge, MA). Photographs were taken using an Olympus BX61 microscope (Olympus Corporation, Center Valley, PA) and Stereo Investigator virtual image tool (MBF Biosciences, Williston, VT). Counting of the number of GFAP positive cells per μm^2^ of field was performed using the Image-Pro Premier software (Media Cybernetics Inc., Rockville, MD).

### Statistical Analysis

Statistical analysis was carried out by Two-way Analysis of Variance followed by Tukey's *post-hoc* test using HSD multiple comparisons (GraphPad Prism 6 software). Values were expressed as mean ± standard error of the mean (SEM). Since the histopathological scoring was done on an ordinal scale, the groups of values were compared by Mann–Whitney *U*-test. A *p* < 0.05 was considered as a significant difference between treatment groups.

## Results

### Blast Exposure Caused Acute Increases in Multiple LPA Species in the Plasma

**Figure 1:**
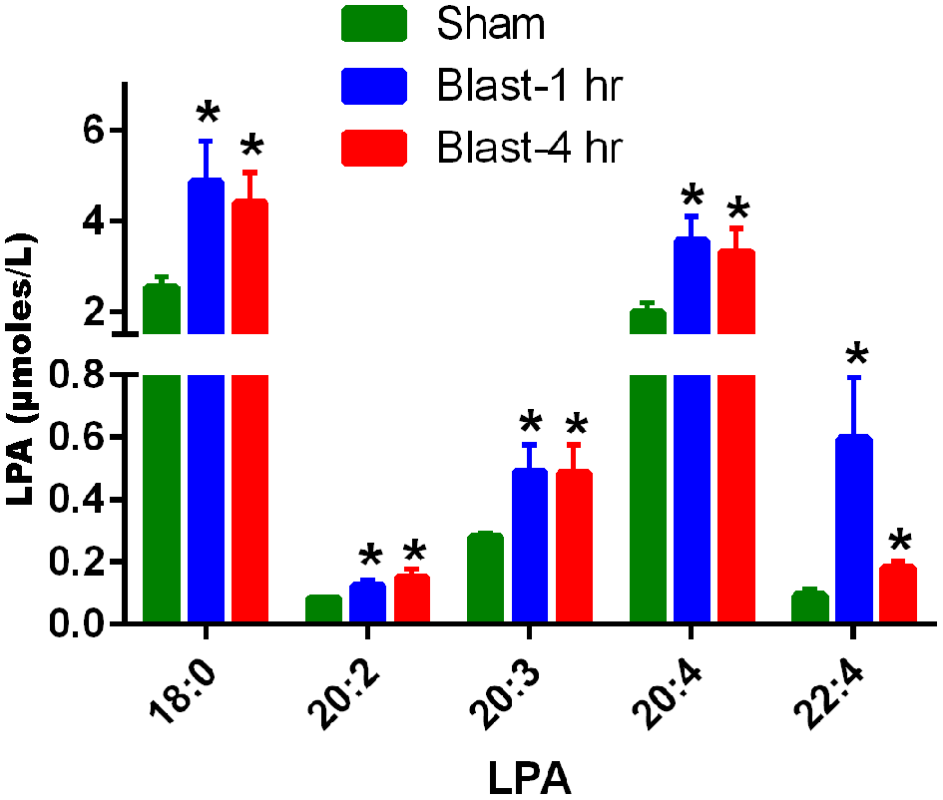
LPA levels in the plasma at 1 and 4 h after blast exposure. The values are expressed in units of μmoles/L as mean ± SEM. Levels of different LPA species in the plasma at 1 and 4 h post-blast are compared to those of sham (*p < 0.05; *n* = 6).

UHPLC and mass spectrometry analysis of the plasma revealed an acute increase in multiple LPA species at 1 and 4 h post-blast exposure. Statistically significant increases in LPA(18:0), LPA(20:2), LPA(20:3), LPA(20:4), and LPA(22:4) in plasma were observed at 1 and 4 h after single blast exposure (Figure 1). Of these, the predominate species were LPA(18:0) and LPA(20:4) although the fold changes after blast exposure were most pronounced for the LPA species measured with poly unsaturated fatty acid substituents. No statistically significant changes in LPA levels were observed between 1 and 4 h post-blast.

### Anti-LPA Antibody Treatment Improved Visual Acuities After Blast Exposure

**Figure 2:**
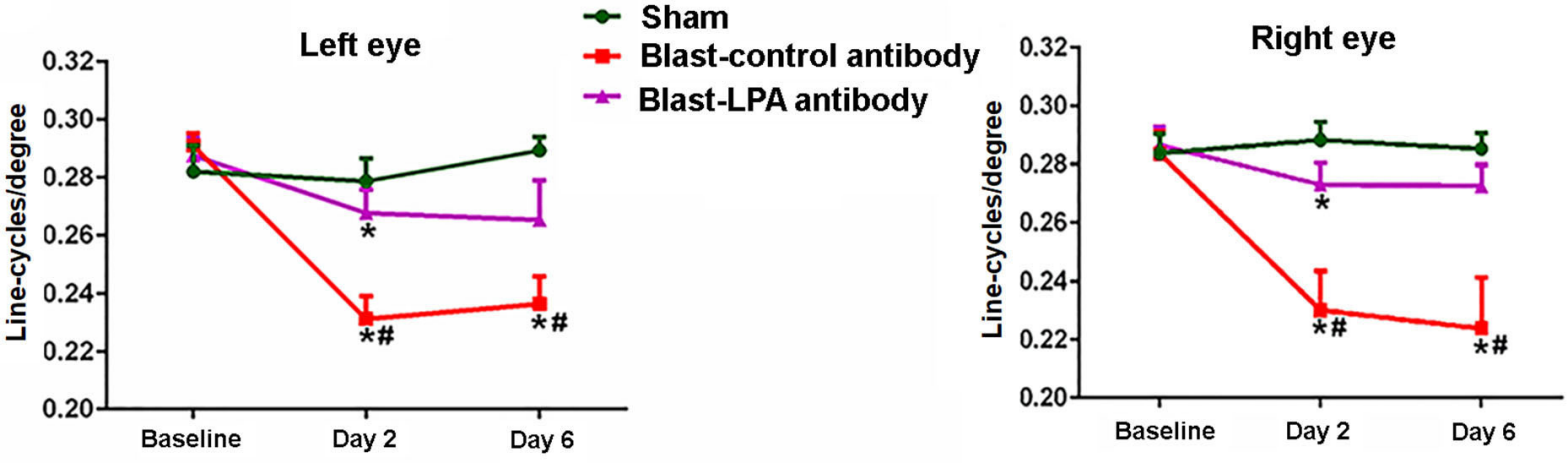
Visual acuities of left and right eyes on days 2 and 6 post-blast. The values are expressed in units of line-cycles/degree as mean ± SEM. Visual acuity values of LPA and control antibody treated blast exposed rats were compared to those of the sham animals (*p < 0.05; *n* = 6). Values of LPA antibody treated rats were compared to those of control antibody treated rats (^**#**^*p* < 0.05).

Visual acuity testing to assess the perception sharpness of vision indicated that a single blast exposure significantly diminished the ability of both eyes to discriminate between optokinetic bar spacing patterns in control antibody-treated rats on days 2 and 6 post-blast, the two time points evaluated (Figure 2). No significant differences in visual acuity were observed in these rats between days 2 and 6 after blast exposure. Compared to sham controls, the anti-LPA antibodies-treated rats showed significantly decreased visual acuity on day 2 and not on day 6. Compared to the isotype-matched control antibody treated rats, the rats that received a single dose of anti-LPA antibodies had significantly higher visual acuity values for both eyes on days 2 and 6 after blast exposure.

### Anti-LPA Antibody Treated Rats Showed Improved Retina Signaling Function After Blast Exposure

**Figure 3:**
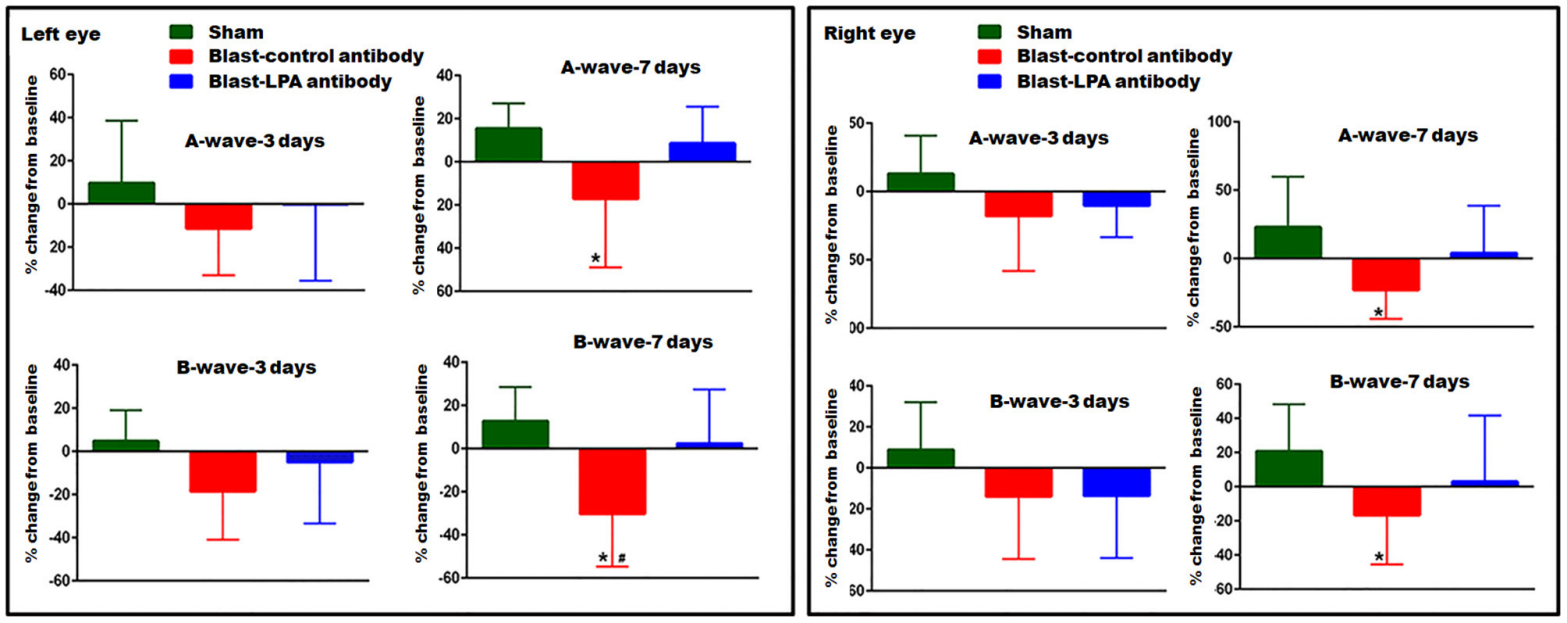
ERG showing A- and B-wave amplitudes of left and right eyes on days 3 and 7 post-blast. Values are expressed in % change from their baseline (prior to blast exposure) as mean ± SEM. A- and B-wave amplitudes of LPA and control antibody treated blast exposed rats were compared to those of the sham animals (*p < 0.05; *n* = 6). Values of LPA antibody treated rats were compared to those of control antibody treated rats (^**#**^*p* < 0.05).

In both eyes, as measured by ERG, A-wave and B-wave amplitudes representative of retina neuronal cell signaling function did not significantly change on day 3 post-blast exposure in control and LPA antibody treated rats (Figure 3). On day 7 post-blast, both A and B-wave amplitudes were decreased in left and right eyes of control antibody treated rats and not in anti-LPA treated rats. Compared to control antibody treated rats, the left eyes of anti-LPA antibody treated rats showed significantly higher B-wave amplitudes. Compared to sham controls, the anti-LPA antibody treated rats did not show significant changes in A or B-wave amplitudes in either eye on days 3 or 7, the two time points evaluated; however, in contrast to the anti-LPA antibody treated rats, measured retina signaling responses never fell below baseline in the sham animals.

### Anti-LPA Antibodies Preserved the Retinal Neuronal Cell Integrity After Blast Exposure

**Figure 4:**
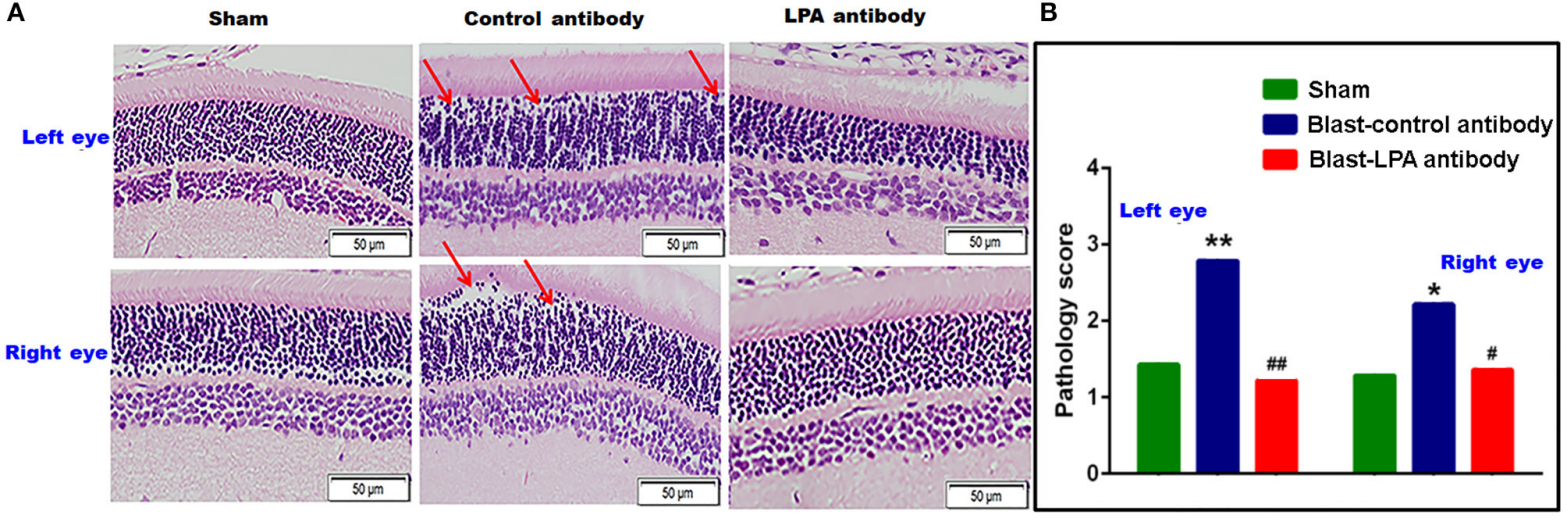
H&E stained retina sections from the left and right eyes on day 8 post-blast. **(A)** Representative pictures of H&E stained retina sections from left and right eyes. Red arrows show the cellular voids in the outer nuclear layer. **(B)** Manual pathology scoring done on the H&E stained retina sections. The values are expressed on ordinal scale of 1–6 as means alone since this representative of non-parametric data. Pathology scores of LPA and control antibody treated blast exposed rats were compared to those of the sham animals (*p < 0.05; **p < 0.01; *n* = 6). Pathology scores of LPA antibody treated rats were compared to those of control antibody treated rats (^**#**^*p* < 0.05; ^**##**^*p* < 0.01).

H&E staining in the retina on day 8 indicated that a single blast exposure affected the integrity of retina neuronal cells in the outer nuclear layer, consisting mainly of rod photoreceptor inner segments (Figure 4). Extensive cellular voids in the outer nuclear layer were observed in both eyes of control antibody treated rats after blast exposure whereas cellular integrity was preserved in anti-LPA antibody treated rats. Compared to sham controls and anti-LPA antibody treated rats, pathological scoring of the H&E stained sections revealed that significant retinal degeneration was present in both eyes of control antibody treated rats. Compared to sham controls, no significant changes in gross pathology of the retina were observed in the anti-LPA antibody treated rats.

### Glial Cell Activation in the Retina After Blast Exposure Was Inhibited by Anti-LPA Antibodies

**Figure 5:**
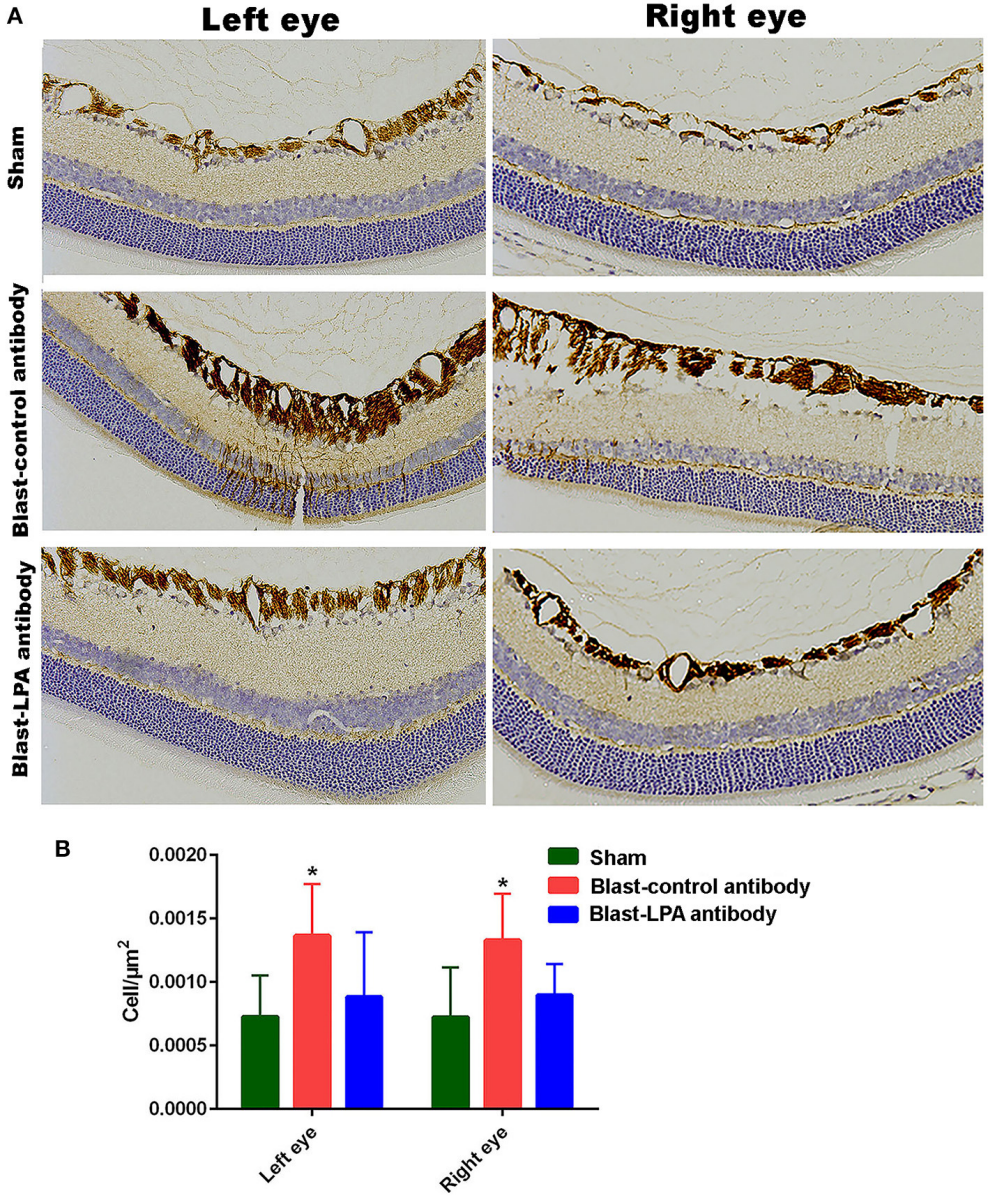
GFAP immunohistochemistry in the retina sections of both eyes on day 8 post-blast. **(A)** Representative pictures of retina sections from left and right eyes after immunohistochemistry with GFAP antibodies. **(B)** The number of activated glial cells expressing GFAP in the retina after blast exposure and treatments. The values are expressed in units of cells/μm^2^ as mean ± SEM. The number of activated glial cells of LPA and control antibody treated blast exposed rats were compared to those of the sham animals (*p < 0.05; *n* = 6).

Immunohistochemical evaluations on day 8 after single blast exposure indicated that significant activation of Müller cells, which then highly express GFAP, occurs in the retina of both eyes of control antibody treated rats, but was not observed in the anti-LPA antibody treated rats (Figure 5). Quantification of glial cell activation by counting of the GFAP positive Müller cells revealed significantly increased retinal injuries to both eyes of control antibody treated rats after blast exposure. In contrast, no increase in the number of activated Müller cells was observed in the eyes of anti-LPA antibody treated rats on day 8 after blast exposure.

## Discussion

The results reported in this study indicate that a single blast exposure can disrupt the integrity of neuronal cells in the eyes, and thus impair normal visual function, and that a single intravenous administration of anti-LPA antibodies immediately after blast exposure can provide significant neuroprotection against this. Amelioration of injury was demonstrated through post-blast improvements in visual acuity (optokinetics), retina signal response to light (ERGs), and retina neuronal cell health (H&E staining and GFAP immunohistochemistry) following anti-LPA treatment. It is possible that the antibody treatment protected other components of the ocular functional system apart from the retina after blast exposure, including the optic nerves, optic chiasm, and optic tracts; hence, further studies are warranted to delineate the particular anatomical constituents underlying these salutary effects. Overall, these findings have positive implications for both military and civilian populations, for whom the incidence of eye injuries and associated visual dysfunctions have increased tremendously with heightened use of improvised explosive devices and other explosive weaponries, and for whom no effective countermeasures have yet been developed. Risks to civilian populations extend beyond weapons and can include blast-induced eye injuries from industrial explosions and related domestic accidents.

As summarized in the introduction, few pre-clinical studies have evaluated blast-induced eye injuries and associated ocular dysfunctions (7–13) and much of this research has been hampered by artifacts confounding the simulations of blast employed in these studies (47). To improve the ecological validity of blast simulations, a state-of-the-art ABS was used for the present study. With a divergent transition section and an end wave eliminator, the ABS eliminates the artifacts of commonly-used constant diameter shock tubes, including end-jet pressures, and provides a means to produce a controlled high fidelity simulation of blast in the laboratory by generating free field Friedlander waveforms quite comparable to field blasts using explosives (42). No prior studies have used an ABS for evaluating the effect of blast overpressure waves on eye injury and associated visual dysfunctions.

Few studies have addressed the effect of LPAs on vision (35–38) and although the levels of LPAs have been shown to increase acutely in the CSF after brain injury (24), no prior studies have examined levels of LPAs in the blood or CSF after blast exposure. Figure 1 illustrates that five different species of LPAs increased significantly in the plasma of rats at 1 and 4 h after a single blast exposure with the greatest fold changes seen in LPA species with polyunsaturated fatty acids. No additional changes were observed between 1 and 4 h, suggesting that the maximum increase occurred immediately after blast exposure and the elevations in LPAs then persisted for at least a few hours. Plasma LPA is predominantly generated by the actions of autotaxin on circulating lyosphospholipids, but platelet activation and thrombosis following blast exposure might contribute to this process. While LPA receptors exhibit some selectivity for LPA species with different fatty acid chain lengths and degrees of unsaturation (48), the biological significance of these longer chain polyunsaturated LPA species is not well understood. However, increases in longer chain/polyunsaturated LPA species have been reported in other settings of inflammation, notably in the lungs (49). We were not able to determine if the LPA changes observed in plasma correspond with increased levels directly inside the retina, which could possibly originate either from activated Müller cells, or by passage from the blood. As with the blood-brain barrier, however, the charged mono-phosphate head group of peripherally generated LPAs would restrict crossing the retinal-pigmented epithelium into the retina, even if produced by nearby choroidal plexus cells, unless there is some disruption of this barrier by the blast shock waves. Transient openings of the blood-brain barrier for up to 72 h do occur in rodent models of blast exposure, resulting from disruption of the capillary endothelial cell tight junction proteins (50). It is also possible that increased blood levels of LPA would trigger the release of circulating pro-inflammatory cytokines for active transport into the retina, as is known for the brain (51), and then promote the blast-induced neuronal injury processes. In this scenario, scavenging LPAs from blood would be very beneficial after eye injuries. In line with this possibility, intravenously injected anti-LPA antibodies would readily bind to LPAs within the blood compartment but would be anticipated to have difficulty accessing LPAs produced directly inside the retina due to the antibody's extremely large molecular size restricting permeation unless if RPE cells were perturbed. We also were not able to determine if the anti-LPA antibodies accumulated inside the intraocular space, i.e., retina or vitreous humor, which would entail the use of rather challenging experimental methods, such as tagged antibodies as a tracer. Figure 5 reveals that glial cell activation occurs in the retina of rats after blast exposure and that a single injection of anti-LPA antibodies reduced the activation of the giant Müller cells, perhaps by suppressing peripheral cytokine production and subsequently preventing secretion of additional LPAs. Thus, the protection of ocular injuries provided by a single injection of anti-LPA antibodies could at least in part be due to tempering the rapid LPA mediated pro-inflammatory activities that promote neurodegeneration directly inside the retina. Retinal ganglion cells express LPA-1 receptors which are known to be involved in triggering their degeneration from oxidative stress (36). LPAs are also able to induce *Tau* protein phosphorylation, which leads to degeneration of neurons through activation of glycogen synthase kinase-3β and protein kinase A (33, 34).

One limitation of the present study is that we have evaluated the efficacy of a single injection and dose of the antibodies. The dose of 25 mg/kg was chosen since the same dose was found to be effective against CCI induced TBI in rodents (24). Preliminary unpublished observations from Lpath, Inc., on a Phase 1 double-blind, placebo-controlled, single ascending dose study to evaluate the safety, tolerability, immunogenicity, and pharmacokinetics of anti-LPA antibodies in healthy volunteers indicated that the antibodies were well tolerated at all doses tested, and no serious adverse events or dose limiting toxicities were observed. The antibodies were administered in five different ascending dose cohorts at 0.5, 1.0, 3.0, 10.0, and 20.0 mg/kg. Study follow-up was completed after dosing with a single intravenous infusion in 36 subjects as planned. The pharmacokinetic profile of anti-LPA antibodies suggests the half-life of the antibody is in the range of 14–18 days, which gives guidance for dosing and treatment regimens in future efficacy studies in patients with eye injuries after blast exposure. Thus, it is likely that the half-life of the antibodies used in the present study was sufficient to preclude repeated dosing. However, it is possible that repeated dosing may provide a more robust protection against blast-induced ocular injuries and associated dysfunctions, if the sequalae of ocular injury is prolonged. A dose-response study with repeated administrations in rats followed by non-human primates is warranted to determine the optimal treatment regimen and identify potential toxicities, if any. Even with a single dosing of anti-LPA antibodies in rats, however, we found significant protection in retinal pathology and associated ocular functions up to 1 week. Although a single systemic dose of the anti-LPA antibody provided significant protection against ocular injury in this model, future pre-clinical and clinical studies could also involve intravitreal injections of antibodies rather than systemic dosing. While the safety profile of systemic dosing of up to 20 mg/kg has been demonstrated in humans (unpublished data from Lpath, Inc.), intraocular administration of therapeutic grade anti-LPA antibodies may be beneficial in neutralizing LPAs produced locally in the eyes after injury. The development of an effective therapy like this will be extremely beneficial to the military population in view of the fact that the incidence of injuries to the ocular system have been increased tremendously in the recent military operations due to the increased use of improvised explosive devices.

In summary visual acuity and ERG assessments (Figures 2, 3) showed significant functional impairments for the eyes of control rats by 3 days post-blast, but a single injection with anti-LPA antibodies at 1 h post-exposure significantly alleviated this impairment, suggesting that the LPA promotion of retinal damage occurs acutely after injury and that early removal of the surge in LPA benefits long-term recoveries. In conclusion, blast exposure triggers the release of different LPA species into the circulation and a single intravenous injection of anti-LPA antibodies at 1 h post-blast exposure, by inactivating one or more pools of LPAs, ultimately leads to a reduction in retina glial mediated neuro-inflammation processes, yielding significant protection against both retinal pathology and ocular dysfunction.

## Data Availability Statement

The original contributions presented in the study are included in the article/supplementary materials, further inquiries can be directed to the corresponding author/s.

## Ethics Statement

The animal study was reviewed and approved by Institutional Animal Care and Use Committee, Walter Reed Army institute of Research.

## Author Contributions

PA, RS, and JL designed the experiments. DW and IG performed the blast experiments. FR performed pathological analyses. JD and AB performed ocular functional tests. AM performed LPA analysis. PA, JD, YW, and JL analyzed the data and wrote the manuscript. All authors contributed to the article and approved the submitted version.

## Conflict of Interest

The authors declare that the research was conducted in the absence of any commercial or financial relationships that could be construed as a potential conflict of interest.

## References

[1] Lemke S Cockerham GC Glynn-Milley C Lin R Cockerham KP Automated perimetry and visual dysfunction in blast-related traumatic brain injury. Ophthalmology. (2016) 123:415–24. 10.1016/j.ophtha.2015.10.003 26581554

[2] Weichel ED Colyer MH. Combat ocular trauma and systemic injury. Curr Opin Ophthalmol. (2008) 19:519–25. 10.1097/ICU.0b013e3283140e98 18854697

[3] Cockerham GC Goodrich GL Weichel ED Orcutt JC Rizzo JF Bower KS. Eye and visual function in traumatic brain injury. J Rehabil Res Dev. (2009) 46:811–8. 10.1682/JRRD.2008.08.0109 20104404

[4] Warden D. Military TBI during the Iraq and Afghanistan wars. J.Head Trauma Rehabil. (2006) 21:398–402. 10.1097/00001199-200609000-00004 16983225

[5] Capo-Aponte JE Urosevich TG Temme LA Tarbett AK Sanghera NK. Visual dysfunctions and symptoms during the subacute stage of blast-induced mild traumatic brain injury. Mil Med. (2012) 177:804–13. 10.7205/MILMED-D-12-00061 22808887

[6] Choi JH Greene WA Johnson AJ Chavko M Cleland JM McCarron RM. Pathophysiology of blast-induced ocular trauma in rats after repeated exposure to low-level blast overpressure. Clin Exp Ophthalmol. (2015) 43:239–46. 10.1111/ceo.12407 25112787

[7] Petras JM Bauman RA Elsayed NM. Visual system degeneration induced by blast overpressure. Toxicology. (1997) 121:41–9. 10.1016/S0300-483X(97)03654-8 9217314

[8] Hines-Beard J Marchetta J Gordon S Chaum E Geisert EE Rex TS. A mouse model of ocular blast injury that induces closed globe anterior and posterior pole damage. Exp Eye Res. (2012) 99:63–70. 10.1016/j.exer.2012.03.013 22504073 PMC3922065

[9] Mohan K Kecova H Hernandez-Merino E Kardon RH Harper MM Retinal ganglion cell damage in an experimental rodent model of blast-mediated traumatic brain injury. Invest Ophthalmol Vis Sci. (2013) 54:3440–50. 10.1167/iovs.12-11522 23620426 PMC4597486

[10] Zou YY Kan EM Lu J Ng KC Tan MH Yao L. Primary blast injury-induced lesions in the retina of adult rats. J Neuroinflammation. (2013) 10:79. 10.1186/1742-2094-10-79 23819902 PMC3707737

[11] Mammadova N Ghaisas S Zenitsky G Sakaguchi DS Kanthasamy AG Greenlee JJ. Lasting Retinal Injury in a Mouse Model of Blast-Induced Trauma. Am J Pathol. (2017) 187:1459–72. 10.1016/j.ajpath.2017.03.005 28606756

[12] DeMar J Sharrow K Hill M Berman J Oliver T Long J. Effects of primary blast overpressure on retina and optic tract in rats. Front Neurol. (2016) 7:59. 10.3389/fneur.2016.00059 27199884 PMC4842954

[13] Jiang Y Liu L Pagadala J Miller DD Steinle JJ. Compound 49b protects against blast-induced retinal injury. J Neuroinflammation. (2013) 10:96. 10.1186/1742-2094-10-96 23899290 PMC3751549

[14] Dutca LM Stasheff SF Hedberg-Buenz A Rudd DS Batra N Blodi FR. Early detection of subclinical visual damage after blast-mediated TBI enables prevention of chronic visual deficit by treatment with P7C3-S243. Invest Ophthalmol Vis Sci. (2014) 55:8330–41. 10.1167/iovs.14-15468 25468886 PMC5102342

[15] Bricker-Anthony C D'Surney L Lunn B Hines-Beard J Jo M Bernardo-Colon A. Erythropoietin either prevents or exacerbates retinal damage from eye trauma depending on treatment timing. Optom Vis Sci. (2017) 94:20–32. 10.1097/OPX.0000000000000898 27281679 PMC5206811

[16] Jiang Y Pagadala J Miller DD Steinle JJ. Insulin-like growth factor-1 binding protein 3 (IGFBP-3) promotes recovery from trauma-induced expression of inflammatory and apoptotic factors in retina. Cytokine. (2014) 70:115–9. 10.1016/j.cyto.2014.07.004 25082650 PMC4319096

[17] Reiner A Heldt SA Presley CS Guley NH Elberger AJ Deng Y. Motor, visual and emotional deficits in mice after closed-head mild traumatic brain injury are alleviated by the novel CB2 inverse agonist SMM-189. Int J Mol Sci. (2014) 16:758–87. 10.3390/ijms16010758 25561230 PMC4307274

[18] Wang HC Choi JH Greene WA Plamper ML Cortez HE Chavko M. Pathophysiology of blast-induced ocular trauma with apoptosis in the retina and optic nerve. Mil Med. (2014) 179:34–40. 10.7205/MILMED-D-13-00504 25102547

[19] Zhu Y Howard JT Edsall PR Morris RB Lund BJ Cleland JM. Blast exposure induces ocular functional changes with increasing blast over-pressures in a rat model. Curr Eye Res. (2019) 44:770–80. 10.1080/02713683.2019.1567791 30947563

[20] Allen RS Motz CT Feola A Chesler KC Haider R Ramachandra Rao S. Long-term functional and structural consequences of primary blast overpressure to the eye. J Neurotrauma. (2018) 35:2104–16. 10.1089/neu.2017.5394 29648979 PMC6098409

[21] Bricker-Anthony C Hines-Beard J D'Surney L Rex TS. Exacerbation of blast-induced ocular trauma by an immune response. J Neuroinflammation. (2014) 11:192. 10.1186/s12974-014-0192-5 25472427 PMC4264554

[22] Eichholtz T Jalink K Fahrenfort I Moolenaar WH. The bioactive phospholipid lysophosphatidic acid is released from activated platelets. Biochem J. (1993) 291(Pt 3):677–80. 10.1042/bj2910677 8489494 PMC1132420

[23] Moolenaar WH Lysophosphatidic acid signalling. Curr Opin Cell Biol. (1995) 7:203–10. 10.1016/0955-0674(95)80029-8 7612272

[24] Crack PJ Zhang M Morganti-Kossmann MC Morris AJ Wojciak JM Fleming JK. Anti-lysophosphatidic acid antibodies improve traumatic brain injury outcomes. J Neuroinflammation. (2014) 11:37. 10.1186/1742-2094-11-37 24576351 PMC3996049

[25] Frugier T Crombie D Conquest A Tjhong F Taylor C Kulkarni T. Modulation of LPA receptor expression in the human brain following neurotrauma. Cell Mol Neurobiol. (2011) 31:569–77. 10.1007/s10571-011-9650-0 21234797 PMC11498475

[26] Ueda H. LPA receptor signaling as a therapeutic target for radical treatment of neuropathic pain and fibromyalgia. Pain Manag. (2019) 10:43–53. 10.2217/pmt-2019-0036 31852400

[27] Srikanth M Chew WS Hind T Lim SM Hay NWJ Lee JHM. Lysophosphatidic acid and its receptor LPA1 mediate carrageenan induced inflammatory pain in mice. Eur J Pharmacol. (2018) 841:49–56. 10.1016/j.ejphar.2018.10.005 30321532

[28] Shano S Moriyama R Chun J Fukushima N. Lysophosphatidic acid stimulates astrocyte proliferation through LPA1. Neurochem Int. (2008) 52:216–20. 10.1016/j.neuint.2007.07.004 17692995 PMC3265988

[29] Holtsberg FW Steiner MR Keller JN Mark RJ Mattson MP Steiner SM. Lysophosphatidic acid induces necrosis and apoptosis in hippocampal neurons. J Neurochem. (1998) 70:66–76. 10.1046/j.1471-4159.1998.70010066.x 9422348

[30] Steiner MR Holtsberg FW Keller JN Mattson MP Steiner SM. Lysophosphatidic acid induction of neuronal apoptosis and necrosis. Ann N Y Acad Sci. (2000) 905:132–41. 10.1111/j.1749-6632.2000.tb06545.x 10818449

[31] Kwon JH Gaire BP Park SJ Shin DY Choi JW. Identifying lysophosphatidic acid receptor subtype 1 (LPA1) as a novel factor to modulate microglial activation and their TNF-alpha production by activating ERK1/2. Biochim Biophys Acta Mol Cell Biol Lipids. (2018) 1863:1237–45. 10.1016/j.bbalip.2018.07.015 30071304

[32] Anliker B Choi JW Lin ME Gardell SE Rivera RR Kennedy G. Lysophosphatidic acid (LPA) and its receptor, LPA1, influence embryonic schwann cell migration, myelination, cell-to-axon segregation. Glia. (2013) 61:2009–22. 10.1002/glia.22572 24115248 PMC3941654

[33] Sayas CL Ariaens A Ponsioen B Moolenaar WH. GSK-3 is activated by the tyrosine kinase Pyk2 during LPA1-mediated neurite retraction. Mol Biol Cell. (2006) 17:1834–44. 10.1091/mbc.e05-07-0688 16452634 PMC1415316

[34] Sun Y Kim NH Yang H Kim SH Huh SO. Lysophosphatidic acid induces neurite retraction in differentiated neuroblastoma cells via GSK-3β activation. Mol Cells. (2011) 31:483–9. 10.1007/s10059-011-1036-0 21499833 PMC3887612

[35] Brault S Gobeil F Jr Fortier A Honore JC Joyal JS. Lysophosphatidic acid induces endothelial cell death by modulating the redox environment. Am J Physiol Regul Integr Comp Physiol. (2007) 292:R1174–83. 10.1152/ajpregu.00619.2006 17122328

[36] Yang C Lafleur J Mwaikambo BR Zhu T Gagnon C Chemtob S. The role of lysophosphatidic acid receptor (LPA1) in the oxygen-induced retinal ganglion cell degeneration. Invest Ophthalmol Vis Sci. (2009) 50:1290–8. 10.1167/iovs.08-1920 18978343

[37] Fincher J Whiteneck C Birgbauer E. G-protein-coupled receptor cell signaling pathways mediating embryonic chick retinal growth cone collapse induced by lysophosphatidic acid and sphingosine-1-phosphate. Dev Neurosci. (2014) 36:443–53. 10.1159/000364858 25138637 PMC4225005

[38] Dacheva I Ullmer C Ceglowska K Nogoceke E Hartmann G Muller S. Lysophosphatidic acids and autotaxin in retinal vein occlusion. Retina. (2016) 36:2311–18. 10.1097/IAE.0000000000001112 27648638

[39] Eisenried A Meidahl ACN Klukinov M Tzabazis AZ Sabbadini RA Clark JD. Nervous system delivery of antilysophosphatidic acid antibody by nasal application attenuates mechanical allodynia after traumatic brain injury in rats. Pain. (2017) 158:2181–8. 10.1097/j.pain.0000000000001019 29028747

[40] Goldshmit Y Matteo R Sztal T Ellett F Frisca F Moreno K. Blockage of lysophosphatidic acid signaling improves spinal cord injury outcomes. Am J Pathol. (2012) 181:978–92. 10.1016/j.ajpath.2012.06.007 22819724 PMC3432439

[41] Heyburn L Abutarboush R Goodrich S Urioste R Batuure A Statz J. Repeated low-level blast overpressure leads to endovascular disruption and alterations in TDP-43 and Piezo2 in a rat model of blast TBI. Front Neurol. (2019) 10:766. 10.3389/fneur.2019.00766 31417481 PMC6682625

[42] Arun P Wilder DM Eken O Urioste R Batuure A Sajja S. Long-term effects of blast exposure: a functional study in rats using an advanced blast simulator. J Neurotrauma. (2020) 37:647–55. 10.1089/neu.2019.6591 31595810

[43] Kraemer MP Mao G Hammill C Yan B Li Y Onono F. Effects of diet and hyperlipidemia on levels and distribution of circulating lysophosphatidic acid. J Lipid Res. (2019) 60:1818–28. 10.1194/jlr.M093096 31484695 PMC6824489

[44] Thomas BB Seiler MJ Sadda SR Coffey PJ Aramant RB. Optokinetic test to evaluate visual acuity of each eye independently. J Neurosci Methods. (2004) 138:7–13. 10.1016/j.jneumeth.2004.03.007 15325106

[45] Prusky GT Harker KT Douglas RM Whishaw IQ. Variation in visual acuity within pigmented, and between pigmented and albino rat strains. Behav Brain Res. (2002) 136:339–48. 10.1016/S0166-4328(02)00126-2 12429395

[46] Kong J Gouras P. The effect of body temperature on the murine electroretinogram. Doc Ophthalmol. (2003) 106:239–42. 10.1023/A:1022988332578 12737500

[47] Needham CE Ritzel D Rule GT Wiri S Young L. Blast testing issues and TBI: experimental models that lead to wrong conclusions. Front Neurol. (2015) 6:72. 10.3389/fneur.2015.00072 25904891 PMC4389725

[48] Kihara Y Mizuno H Chun J. Lysophospholipid receptors in drug discovery. Exp Cell Res. (2015) 333:171–7. 10.1016/j.yexcr.2014.11.020 25499971 PMC4408218

[49] Ackerman SJ Park GY Christman JW Nyenhuis S Berdyshev E Natarajan V. Polyunsaturated lysophosphatidic acid as a potential asthma biomarker. Biomark Med. (2016) 10:123–35. 10.2217/bmm.15.93 26808693 PMC4881841

[50] Logsdon AF Meabon JS Cline MM Bullock KM Raskind MA Peskind ER. Blast exposure elicits blood-brain barrier disruption and repair mediated by tight junction integrity and nitric oxide dependent processes. Sci Rep. (2018) 8:11344. 10.1038/s41598-018-29341-6 30054495 PMC6063850

[51] Banks WA Kastin AJ Broadwell RD. Passage of cytokines across the blood-brain barrier. Neuroimmunomodulation. (1995) 2:241–8. 10.1159/000097202 8963753

